# Novel chromaticity similarity based color texture descriptor for digital pathology image analysis

**DOI:** 10.1371/journal.pone.0206996

**Published:** 2018-11-12

**Authors:** Xingyu Li, Konstantinos N. Plataniotis

**Affiliations:** Multimedia Lab, The Edward S. Rogers Department of Electrical and Computer Engineering, University of Toronto, Toronto, Ontario, Canada; Institute of Automation Chinese Academy of Sciences, CHINA

## Abstract

Pathology images are color in nature due to the use of chemical staining in biopsy examination. Aware of the high color diagnosticity in pathology images, this work introduces a compact rotation-invariant texture descriptor, named quantized diagnostic counter-color pattern (QDCP), for digital pathology image understanding. On the basis of color similarity quantified by the inner product of unit-length color vectors, local counter-color textons are indexed first. Then the underlined distribution of QDCP indexes is estimated by an image-wise histogram. Since QDCP is computed based on color difference directly, it is robust to small color variation usually observed in pathology images. This study also discusses QDCP’s extraction, parameter settings, and feature fusion techniques in a generic pathology image analysis pipeline, and introduces two more descriptors QDCP-LBP and QDCP/LBP. Experimentation on public pathology image sets suggests that the introduced color texture descriptors, especially QDCP-LBP, outperform prior color texture features in terms of strong descriptive power, low computational complexity, and high adaptability to different image sets.

## Introduction

Pathology is a medical sub-specialty that studies and practices the diagnosis of disease through examining biopsy samples or surgical specimens under microscopes by pathologists. It serves as the golden truth of cancer diagnosis. To address subjectivity in pathology examination [1, 2], digital pathology exploits image analysis techniques and pattern recognition algorithms for histological information understanding in tissue images, and merges as a promising approach owing to its time-efficiency, consistency, and objectivity. Essentially, a digital pathology diagnosis system is a pattern recognition system. Given a query pathology image, a machine understands it by comparing a set of quantitative features from the image against the stored feature sets in the database. Hence, extraction of discriminative features from color pathology images is important.

Texture is one of most significant information sources in pathology image analysis. As early as 2004, a study reported that texture features could distinguish among normal tissue areas and cancerous areas, providing a means of locating morbid regions in pathology images [3]. Later, textures described by co-occurrence matrices, steerable filters, and fractal analysis are included for prostate cancer detection and breast cancer classification [4–6]. Image filtering based texture descriptors together with nuclei’s morphology features are used for mitotic detection in breast cancer images [7]. Recently, local binary patterns (LBP) [8] is demonstrated to be discriminative in lymphoblasts classification [9, 10].

Due to the use of chemical stains for histological substance’s highlight, color information provides insight to pathology image understanding. In [11, 12], the authors compared grayscale texture features to their color-version texture descriptors in pathology image classification, and concluded that color texture descriptors improved classification performance when limited appearance variation resulting from the disagreement of illumination conditions existed in pathology images. In the study of breast cancer pathology image diagnosis [13], the state-of-the-art vector processing based texture descriptor, named LCVBP [14], was adopted to classify normal and malignant breast biopsy images, achieving 87.51% classification accuracy. This features is also exploited by a recent histological image classification study [15]. A circular Hue-LBP descriptor, denoted by CHLBP, was demonstrated to obtain promising results on pathology image texture analysis [16]. It should be noted that except the CHLBP, all of other color texture descriptors used in pathology image analysis literature were proposed for other applications and thus have various limitations on quantitative description of pathology images. For instance, variation in tissue substances’ organization is informative for cancer diagnosis. Due to multi-staining in pathology images, texture patterns composed of counter colors are very descriptive for inter-substances’ spatial arrangement. As most color texture descriptors are computed on the basis of color signal orders, they are sensitive to small color variation usually observed in the same type of stained histological elements, and thus not descriptive for counter-color textures in pathology images. To clarify, in this paper, color texture is a term to describe color content in an image, providing information on image color spatial organization.

This work exploits counter-color information in pathology images and proposes a compact rotation-invariant color descriptor, named quantized diagnostic counter-color pattern (QDCP), for histological texture composed of different stained tissue substances in pathology image. The motivation behind relies on the close connection between color diagnosticity [17] and stained tissue substances in pathology images. Chemical dyes are used to highlight histological components of interest in pathology. As a result, colors’ spatial distribution is a strong indicator of spatial organization of tissue substances in images, and descriptors based on counter-color distribution are insightful in pathology image analysis. As small color variation usually exists in pathology images, to address this imperfection, QDCP adopts a thresholding method to measure whether two color vectors should be considered as counter colors. Specifically, to alleviate the influence of image brightness on QDCP, the proposed descriptor is computed from color vector’s orientation. This is because color is quantified by a multi-variant vector, each element representing the value in one color channel. The magnitude of the vector represents the brightness of a color, and the vector’s orientation correlates well with color’s chromaticity [18, 19]. To compute QDCP, rotation-invariant local color textons are indexed in a texture structural analysis manner first and then the occurrence of various textons is summarized in an image-wise histogram. It is noteworthy that different from most previous color texture analysis that compute color texture patterns from channel pairs, QDCP is directly extracted from color vectors, where color vectors’ similarity is quantified by angular differences between color vector’s orientations. We want to point out that though QDCP’s construction seems similar to certain texture features, its motivation and focus on counter-color texture representation are novel and distinct.

In addition, we elaborate the computation of the QDCP descriptor in a generic pathology image analysis framework. Parameter settings and texture feature fusion techniques are discussed. Since QDCP contains complementary information to classic grayscale texture descriptors, two numerical features, QDCP-LBP and QDCP/LBP, are introduced for complete color texture representation. Experimentation demonstrates that the proposed descriptors greatly boost the performance of texture analysis in pathology images and always achieve top performance in different image sets. It is noteworthy that the high adaptability of the proposed QDCP-LBP feature to different datasets is very attractive in image description. It is true that one can use a specially-designed analysis tool for good performance if images’ property and content are known beforehand. However generally, such information is not well known. Therefore, an analysis tool having such high adaptability is highly demanding in image analysis.

**To summarize, the main contribution of this work are**:

1. Motivated by high color diagnosticity in pathology images, we propose the use of counter color’s spatial arrangement to characterize histological content composed of different stained tissue substances. To the best of our knowledge, this innovation is first explicitly explained and presented in color texture analysis literature, and provides a new vision to counter-color analysis in pathology images.
2. A rotation-invariant color texture descriptor, QDCP, is introduced. It is advanced in counter-color content description for pathology image analysis. In a very compact form, QDCP is able to achieving better, or at least comparable, classification performance to the state-of-the-arts in different pathology image sets.
3. Two numerical color texture descriptors, QDCP-LBP and QDCP/LBP, are introduced based on different feature fusion techniques. With smaller computation complexity, experimentation indicates that the proposed texture features, especially QDCP-LBP, are capable of always achieving top performance in different pathology image analysis tasks. Such high adaptability enables QDCP-LBP to be a competitive descriptor in various image analysis tasks.

The rest of this paper is organized as follows. In Section of Prior Arts, the state-of-the-art color texture analysis methods are briefly reviewed. Section Methods specifies the construction of the proposed QDCP descriptor, and elaborates the computation of QDCP from pathology images in a general digital pathology pipeline. Experimental Design with applications of pathology images and the results are presented in Section Experimental Design and Results and Discussion, respectively. Finally, conclusions are drawn in the last section.

## Prior arts

Color texture is informative in image analysis. However, compared to the well-studied gray-scale texture analysis, research on color texture representation relatively falls behind. This may be attributed to the multivariate nature of color signals. To address the vectorial nature of color signals, currently, three categories of color texture quantification methods are considered in literature.

The first paradigm applies traditional scalar texture descriptors to image channels separately, each individual channel of a color image being considered as a monochrome image [11, 20–23]. On one hand, independently processing color components ignores the high correlation that exists between color channels. Hence the obtained texture descriptors may have abundant information redundancy, resulting in less compact descriptors. On the other hand, this paradigm may lose information about interactions between channels, leading to ineffective features. To obtain a compact color texture descriptor, the LBP operator is applied to the hue channel directly [24]. The obtained feature achieves good performance on the PASCAL visual object classes challenge 2007 image benchmark. To further address the circular nature of hue in color texture representation, a circular Hue-LBP (in short, CHLBP) [16] is proposed based on the angles between hue values. As the hue component is ill-defined for achromatic colors, the proposed Hue-LBP is unreliable in image areas containing a large amount of achromatic pixels.

The second category of algorithms considers color inter-channel dependency in texture feature extraction. In the opponent color (OC) texture description method [25], authors propose to apply scalar texture analysis methods to each pair of color channels for color texture quantification. For example, to compute OC-LBP from an RGB-format image, 6 LBP histograms are extracted from the red-red, green-green, blue-blue, red-green, red-blue, and green-blue channel pairs. But this method is computationally intensive. Instead of examining texture patterns within channel pairs, an image indexing study introduces a 3-D co-occurrence matrix to summarize the joint distribution of LBP in the red, green, and blue channels for an RGB image [26]. Although the so-called joint-LBP is demonstrated outperforming OC-LBP in endoscopic image classification [27], the disadvantage of joint-LBP is the large size of the color co-occurrence matrix whose elements may become sparse and unstable.

The last category of methods treats a color signal as a vector and quantifies color textures using vector processing. Norm-LBP is extracted from color vector’s magnitude in the RGB or CIELAB color space [27]. Shortly, the local color vector binary patterns (LCVBP), composed of 3 angular texture patterns and the Norm-LBP, is proposed for face recognition [14]. In this work, the color angular patterns are quantified by applying the LBP operator to the relative phases within channel pairs in the YIQ color space. Due to high correlations between color channels, the three angular pattern descriptors may have information redundancy. To obtain a compact descriptor, a face expression recognition study introduces J-LCVBP, which adopts the sin distance [28] to measure similarity between two color vectors and uses of the J-th largest sin distance as a threshold to construct a LBP descriptor within a neighborhood [29]. Though J-LCVBP is good at addressing micro skin color difference associated with different expressions on human faces, the over-emphasis on small color variation in image smooth areas makes it less effective to describe counter-color textures in pathology images. Local color contrast (LCC) makes the use of angular difference to quantize color contrast statistics between a center pixel and the local mean color derived from its neighborhood [30]. LCC is an analogy to the local intensity standard deviation in a grayscale image, and irrelevant to local color texton description. To resist the color changes of an illuminant, local angular patterns (LAP) are extracted from the red-green, red-blue, and green-blue channel pairs in the RGB color space [31]. For each channel pair, angular differences between center pixels and their local means are quantized and fed to the LBP framework to generate a LAP. Due to high correlations between color channels, the 3 LAP descriptors are not compact and may have information redundancy.

The state of the art of color texture analysis algorithms with the proposed texture descriptor QDCP are summarized in Table 1. Note that though the vector-based color texture analysis methods are demonstrated more effective for color image analysis, they have different limitations as discussed above. Besides, as existing methods are based on color signal order statistics, which is sensitive to small color variation, they are not stable to represent medical information in terms of counter-color histological substances in pathology images.

**Table 1:** Comparison of the proposed versus previous color texture descriptors.

Ref.	Name	Treatment of color channels	Basic operation for texton description	Length
[12]	Ind-LBP^2^	Independent	Marginal ordering [19] of color vectors	3*N*
[24]	Hue-LBP	Independent	Linear ordering of hue	*N*
[16]	CHLBP	Independent	Angular similarity of hue	*N*
[25]	OC-LBP	Joint	Marginal ordering of color vectors	6*N*
[26]	Joint-LBP	Joint	Marginal ordering of color vectors	*N*^2^
[27]	Norm-LBP	Vectorial	Reduced ordering [19] of color vectors	*N*
[14]	LCVBP	Vectorial	Reduced ordering of color vectors	4*N*
[29]	J-LCVBP	Vectorial	Reduced ordering of color vectors	2*N*
[30]	LCC	Vectorial	Contrast of color angular difference	256
[31]	LAP	Vectorial	Reduced ordering of color vectors’ similarity	3*N*
Proposed	QDCP	Vectorial	Angular similarity of color vectors’ orientations	*N*

Since all texture analysis methods in this table are variants of LBP, *N* in the table represents the length of a LBP histogram generated with *log*_2_ *N*-pixel neighborhood. Ind-LBP is short for the method that applies LBP to each color channel separately, and it is listed in this table as an typical example in the category of independent color texture analysis paradigm.

## Methods

In this section, we first introduce the novel color similarity based texture descriptor, called quantized diagnositc counter-color patters (QDCP). It is proposed for effectively coding medical information conveyed by counter colors in pathology images. Then related issues about applying QDCP to pathology image analysis are discussed. To clarify, vector variables in this work are denoted in boldface.

### Quantized diagnostic counter-color patterns

In brief, given a color image **I**, its orientation image **I**_*O*_ is generated using color’s magnitude-direction (MD) representation [19] (or the so-called brightness-chromaticity (CB) model in literature). Based on predetermined neighborhood Ω_*N*_ and threshold *T*_*O*_, various local counter-color texton in **I**_*O*_ are indexed, forming a scalar image *I*_*QDCP*_. Finally, the statistics of local color texton are summarized by an image-wise histogram *H*_*QDCP*_, which is then used as a color texture feature in analysis. It is noteworthy that the introduced descriptor QDCP combines both structural and statistical texture analysis approaches. On one hand, a texton which represents spatial placement of counter-color elements in a small neighborhood is analyzed and indexed. On the other hand, the obtained histogram approximates the distribution of texton patterns in an image. Details about the introduced color texture descriptor is represented as follows.

#### MD representation of color images

A color image is represented by a function **I**: **Z**^2^ → **Z**^3^ that maps a pixel *p* = (*x*, *y*) in the 2-dimensional image plane to a 3-dimensional vector **I**(*p*) = [*i*_1_, *i*_2_, *i*_3_]^*T*^ in a color space. For instance, in the RGB color model, *i*_1_, *i*_2_, and *i*_3_ correspond to the red, green, and blue components, respectively. Since color vector’s elements are highly correlated, chroma information is more closely related to the relative values among color channels, and color vector’s orientation/direction correlates well with color description [18, 32–34]. Hence, to describe spatial arrangement of image elements in counter colors, we propose to extract the descriptor from color vectors’ orientations.

Specifically, any color vector **I**(*p*) = [*i*_1_, *i*_2_, *i*_3_]^*T*^ can be uniquely described by its magnitude *I*_*M*_(*p*) and orientation **I**_*O*_(*p*) in color magnitude-directional representation [19]:

$$\begin{array}{ccc} & & I\left(p\right)=I_{M}\left(p\right)\times I_{O}\left(p\right)\\ & \text{where} & \left\{ \begin{array}{c} I_{M}\left(p\right)=\sqrt{{\left(i_{1}\right)}^{2}+{\left(i_{2}\right)}^{2}+{\left(i_{3}\right)}^{2}}\\ I_{O}\left(p\right)=\frac{I(p)}{I_{M}\left(p\right)},\;\text{s}.\text{t}.\|I_{O}\left(p\right)\|=1 \end{array}.\right. \end{array}$$ (1)

**Fig 1:**
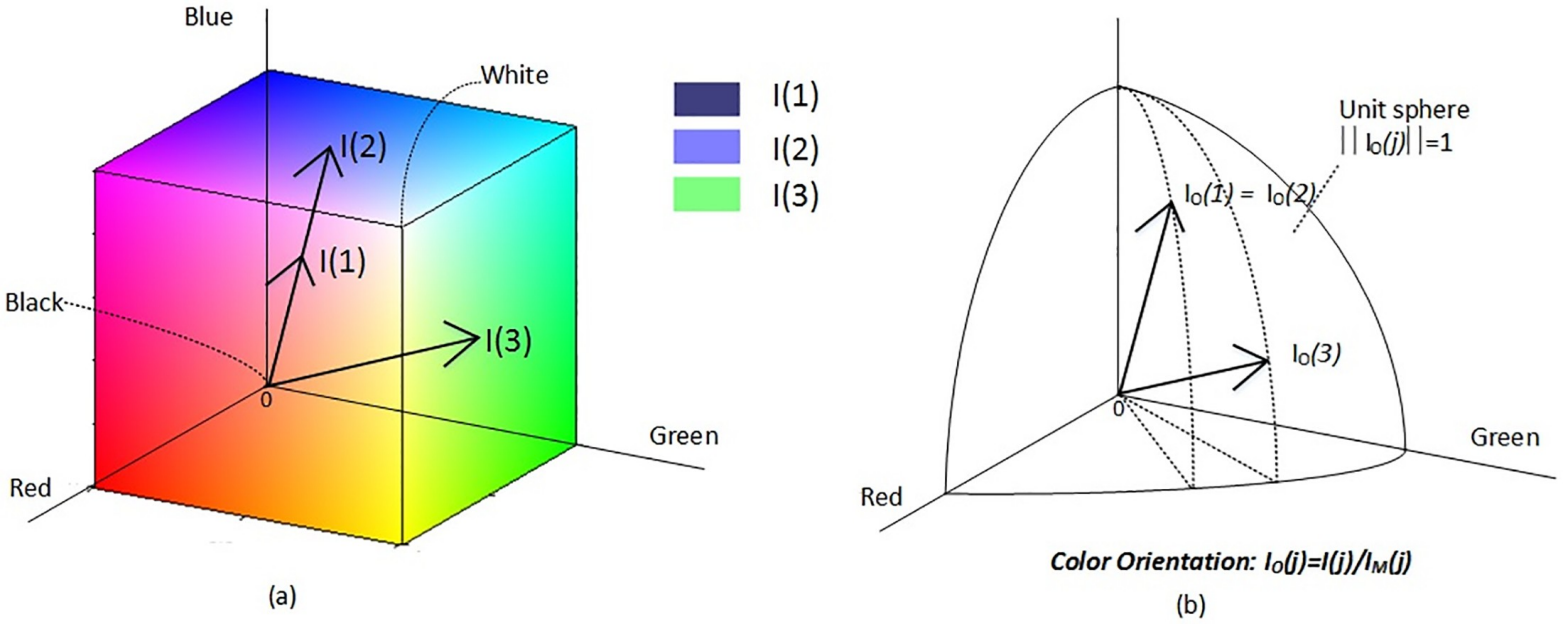
An example of the color orientation components in the MD representation in the RGB color space. (a) An RGB color cube with three color vectors, and (b) their orientations on the unit sphere.

In (1), different from image magnitude *I*_*M*_(*p*) which is a scalar variable, the direction of a color vector is defined by a unit-length vector **I**_*O*_(*p*) = [*i*_1_/*I*_*M*_(*p*), *i*_2_/*I*_*M*_(*p*), *i*_3_/*I*_*M*_(*p*)]^*T*^ in the vector field. Since **I**_*O*_(*p*) is a self-contained directional variable on the chromatic unit sphere, analysis of color texture based on it is also simplified and restricted on the unit sphere. Fig 1 depicts three color vectors and their orientations in the RGB color cube, where the co-linear **I**(1) and **I**(2) have chromaticity of blue, while **I**(3) that points to another direction corresponds to green.

#### Metric of color similarity in **I**_*O*_

Since **I**_*O*_(*p*) is a directional variable on the unit sphere, adopting linear operations to **I**_*O*_(*p*) for color texture quantification is inappropriate. Hence, we propose the use of color angular difference/similarity as the basic metric to construct the proposed color texture descriptor. Mathematically, the central angle between the two chromaticity vectors **I**_*O*_(*p*) and **I**_*O*_(*i*) in radians is

$$\begin{array}{c} ∠\left(I_{O}\left(p\right),I_{O}\left(i\right)\right)=\arccos\left(\frac{I_{O}\left(p\right)I_{O}{\left(i\right)}^{T}}{\left\|I_{O}\left(p\right)\|\|I_{O}\left(i\right)\right\|}\right), \end{array}$$ (2)

where **I**_*O*_(*p*)**I**_*O*_(*i*)^*T*^ denotes the inner product of the two vectors. As ‖**I**_*O*_(*p*)‖ = ‖**I**_*O*_(*i*)‖ = 1, and arccos(⋅) is monotonously decreasing for ∠(**I**_*O*_(*p*), **I**_*O*_(*i*)) ∈ [0, 180],

$$\begin{array}{c} ∠\left(I_{O}\left(p\right),I_{O}\left(i\right)\right)\propto I_{O}\left(p\right)I_{O}{\left(i\right)}^{T}. \end{array}$$ (3)

To avoid the inverse trigonometric operation which is computational intensive, the inner product **I**_*O*_(*p*)**I**_*O*_(*i*)^*T*^ is used to measure color similarity in this work. For ease of reference, in this work

$$\begin{array}{c} m_{p,i}=I_{O}\left(p\right)I_{O}{\left(i\right)}^{T}. \end{array}$$ (4)

A small *m*_*p*,*i*_ indicates two different color orientations, and *m*_*p*,*i*_ = 1 implies that two color vectors are co-linear.

#### Local texton indexing based on color similarity

We first consider color spatial distribution in a small image patch. For clarification, given a color orientation image **I**_*O*_, a small patch centered at a pixel *p* is composed by its *N* neighborhood pixels within a predefined area Ω_*N*_. In this work, to obtain rotation-invariant descriptor, the classical circular neighborhood is taken to define Ω_*N*_. For instance, given a center pixel, Ω_8_ consists of 8 equally spaced pixels on a circle of unit radius around it, under which there are 36 different rotation-invariant patterns. It should be noted that in the circle neighborhood, there must be some neighbor points not on integer coordinates. Color vectors for these point are computed using the bilinear interpolation based on the nearest 4 pixels on image integer coordinates. The assumption behind this operation is that image color is smooth and without abrupt change.

For each pixel *p* in **I**_*O*_ whose neighbors are all within an image, a vector **M**(*p*) = {*m*_*p*,*i*_, *i* ∈ Ω_*N*_} is generated to quantify local color differences between the center pixel **I**_*O*_(*p*) and its neighbor pixels **I**_*O*_(*i*) for **i** ∈ Ω_*N*_. That is, **M**(*p*) provides information on local color similarity with respect to the center pixel.

Note that the real-value quantity *m*_*p*,*i*_ is sensitive to small color variation which may be introduced by inconsistent staining depths, imaging noise, or uneven illumination. To obtain a robust descriptor against small color variation, a binarization *b*_*p*,*i*_ ∈ {0, 1} with respect to a threshold *T*_*O*_ is introduced to determine whether color difference *m*_*p*,*i*_ is smaller enough to distinguish image colors. To be more precise, *b*_*p*,*i*_ = 1 for *m*_*p*,*i*_ < *T*_*O*_, which suggests a major color change due to different stained substances in images. With the obtained similarity vector **M**(*p*) and threshold *T*_*O*_, a value *I*_*QDCP*_(*p*) is generated to represent the rotation-invariant local counter-color pattern around pixel *p* as follows:

$$\begin{array}{ll} I_{QDCP}\left(p\right) & =\underset{0\leq n<N}{\text{min}}\left\{\sum_{i\in \Omega_{N}}b_{p,i}\times 2^{\left[\left(i+n\right)\;\;\text{mod}\;N\right]}\right\},\\ \text{where} & b_{p,i}=\{\begin{array}{ll} 1 & \text{if}\;m_{p,i}<T_{O}\\ 0 & \text{otherwise} \end{array}, \end{array}$$ (5)

Note the min(⋅) together with the mod (⋅) operations in (5) ensure QDCP’s rotation-invariant property. Since a tissue slide may be placed along any orientation during imaging, the property of rotation invariance is of paramount significance in pathology image analysis.

The binarization threshold *T*_*O*_ can be either predetermined or adaptively set based on applications. It is noteworthy that for the same image, different *T*_*O*_ leads to distinct color texture descriptors. Briefly, fine textures associated with small color change can be extracted with a large *T*_*O*_, and a smaller threshold on the color similarity vector results in a coarse color texture descriptor. We discuss the parameter setting of *T*_*O*_ particularly in pathology image analysis in the next section.

#### Summary of texton statistics

From the viewpoint of texture statistics analysis approaches, texture is considered as a probabilistic generator of texton and the underlying probability distribution of texton can be used for image abstract representation. Following the histogram of equivalent patterns (HEP) paradigm [35], an image-wise histogram *H*_*QDCP*_, the approximation of color textons’s distribution, is produced from the scalar image *I*_*QDCP*_ for image characterization. Mathematically, the value in the *j*^*th*^ bin in *H*_*QDCP*_ is

$$\begin{array}{ccc} H_{QDCP}\left(j\right) & = & \sum_{p}\delta \left(I_{QDCP}\left(p\right),j\right), \end{array}$$ (6)

where *δ*(⋅) is the Dirac delta function. It is noteworthy that since QDCP is computed based on color difference which is less sensitive to image luma components, it is robust to vignette and inconsistent illumination in pathology images.

### Pathology image analysis using QDCP

#### Overview of color texture based analysis pipeline

**Fig 2:**
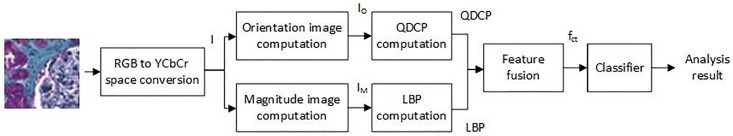
QDCP based pathology image color texture analysis pipeline used in this work.

Fig 2 depicts pathology image analysis workflow in this work. Given a color pathology image **I**, it is first converted to the YCbCr color space, a linear transformation of the RGB domain in (7).

$$\begin{array}{c} (\begin{array}{c} Y\\ Cb\\ Cr \end{array})=(\begin{array}{ccc} 0.299 & 0.587 & 0.114\\ 0.596 & -0.274 & -0.322\\ 0.211 & -0.523 & 0.312 \end{array})\;(\begin{array}{c} R\\ G\\ B \end{array}). \end{array}$$ (7)

We select the YCbCr model to extract the QDCP descriptor for reasons as follows. First, in the YCbCr domain, the color similarity metric in (4) is more effective to distinguish stain colors in pathology images. For instance, in Masson’s trichrome stained pathology images, collagen and mucus appear in green-blue colors, while muscle and cytoplasm are in red. In the RGB color space, all colors in the green-blue plane are orthogonal to red, which implies that obtained color similarity metric is 0 between green-blue colors and red. In contrast, after above non-orthonormal transformation in (7), this issue is solved and the similarity metric has more different values, which facilitates distinguishing histological objects in pathology images using QDCP. Second, the performance of QDCP depends on the selection of color difference threshold *T*_*O*_. In the RGB color space, a color vectors is valid with positive vector components in a 3-D Cartesian coordinate system, as depicted in Fig 1(a). Consequently, **I**_*O*_(*p*) locates on the positive eighth of the unit sphere, and *T*_*O*_ has to be the in the range of [0, 1]. In contrast, in the YCbCr color space where Cb/Cr components can be negative, the corresponding **I**_*O*_(*p*) occupies most of the upper sphere. Hence for the same images, the threshold range expands to [−1, 1], which greatly alleviates QDCP’s sensitivity to the threshold setting. In experimentation, the YCbCr space is compared to other color spaces (RGB, CIELab, and I1H2H3) that are often used in pathology image analysis. The results indicate that YCbCr domain always reaches the top performance in all examined image sets.

Then the YCbCr-format image is decomposed into orientation image **I**_*O*_ and magnitude image *I*_*M*_ following (1), from which QDCP and LBP are extracted to form a long color texture feature *f*_*ct*_ using different feature fusion techniques. Note, as QDCP describes color texture patterns in the orientation image only, texture patterns in the magnitude image should also be included in analysis. Given *I*_*M*_(*p*) being a linear variable, we select LBP to characterize content in the magnitude image due to the descriptive power of LBP. We want to point out that in the YCbCr domain, *I*_*M*_/**I**_*O*_ decomposition is superior to the Luminance/Chromaticity separation (corresponding to Y-axis/CbCr-plane) in pathology image analysis for following reason. In the YCbCr color space, CbCr components are ill-defined when the vector is close to an achromatic color. Consequently, color analysis on the CbCr plane may be problematic, especially for pathology images which usually contains achromatic background areas. The decomposition of *I*_*M*_/**I**_*O*_ can avoid this problem as an achromatic color in the YCbCr color space has a well-defined orientation, which can be denoted by a unit vector [1, 0, 0].

Finally, a classifier is applied to the long feature vector *f*_*ct*_ to obtain image analysis result.

Note that color variation is often observed in pathology images [36] and color texture features are reliable when examined pathology images have limited color variation [11, 12]. Hence, in this paper, we assume that such appearance variation, if it exists, has been removed beforehand by color normalization approaches [37–40].

#### Selection of threshold *T*_*O*_ for QDCP

**Fig 3:**
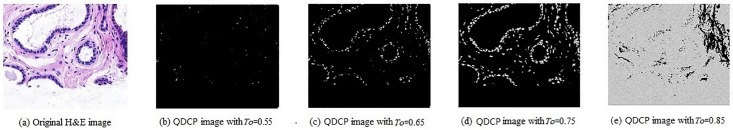
QDCP images generated under different thresholds *T*_*O*_ with 8-point unit-radius circular neighborhood. Given a normal breast biopsy image (a) from the UCSB breast cancer dataset [41], a small threshold results in a QDCP image with large black area as most color changes are considered insignificant for image description (b); whereas with a large threshold, QDCP is sensitive to small color variations, and the major color change may be overwhelmed by small color variations (d). Since there are 36 rotation-invariant patterns under the setting, all QDCP images are normalized with respect to 36 for visualization.

To quantitatively represent diagnostic counter-color patterns by QDCP, an appropriate threshold *T*_*O*_ is vital. Generally, a small threshold leads to a less-descriptive feature, while a very large *T*_*O*_ makes QDCP sensitive to small color change introduced by imaging noise or non-uniform imaging illumination. Fig 3 presents examples of *I*_*QDCP*_ with respect to different thresholds. In the figure, the QDCP image computed with a smaller threshold, *T*_*O*_ = 0.55, has fewer details, as most color differences are not high enough to be considered as major color changes. However, with a larger threshold, for instance, *T*_*O*_ = 0.85, most color variations in the image are significant with respect to *T*_*O*_ and hence contributory to image description. Consequently, the obtained QDCP image shown in Fig 3(d) is less descriptive for nuclei distribution in the image.

It should be noted that selection of the threshold for a QDCP descriptor is application dependent. It is non-trivial to set the optimal threshold by a generic algorithm. So in this study, we provide several strategies to select *T*_*O*_ for QDCP’s construction in pathology image analysis. First, if prior knowledge about chemical dyes is known, a value which is slightly larger than the inner product of corresponding stains’ color vectors can be used as *T*_*O*_. By using this threshold, QDCP is capable of extracting information about diagnostic counter-color patterns composed of stained histological components. Otherwise, we believe *T*_*O*_ ∈ (0.75 − 0.85), which corresponds to colors separating around 30–40 degrees, is a good start to compute QDCP from the YCbCr domain. Experimentation later demonstrates that *T*_*O*_ ∈ (0.75 − 0.85) is capable of generating considerably good classification in different datasets. In addition, the various QDCP descriptors generated under different thresholds can be combined together, so that both coarse and fine color textures are taken into consideration for image understanding.

#### QDCP and LBP feature fusion

QDCP and LBP represent image textures in the orientation image and the magnitude image, respectively. To form a complete color texture feature, we can combine QDCP and LBP by concatenation or joint manner.

- In the concatenation manner, we compute individual histograms of QDCP and LBP indexes. Then the two histograms are concatenated together. In this work, the concatenation fusion is represented by QDCP-LBP.
- In the joint fusion method, a joint 2-dimensional histogram is generated, where co-occurrence of QDCP and LBP indexes is recorded. Then the 2-dimensional histogram is vectorized to form a long feature vector. The joint fusion manner is represented by QDCP/LBP in this paper.

Compared to the joint fusion method which generates a long feature vector, the concatenation manner is computationally efficient with the penalty of discarding the co-occurrence information between QDCP and LBP.

## Experimental design

In this section, experimental design with applications in pathology image analysis, which includes information about testing pathology image sets and evaluation methodology, is specified.

### Pathology image sets

This study takes two public pathology image sets, the GlomDB glomeruli dataset [12] and the ALL-IDB2 dataset [42], as experimental data to evaluate the proposed texture descriptor.

**Fig 4:**
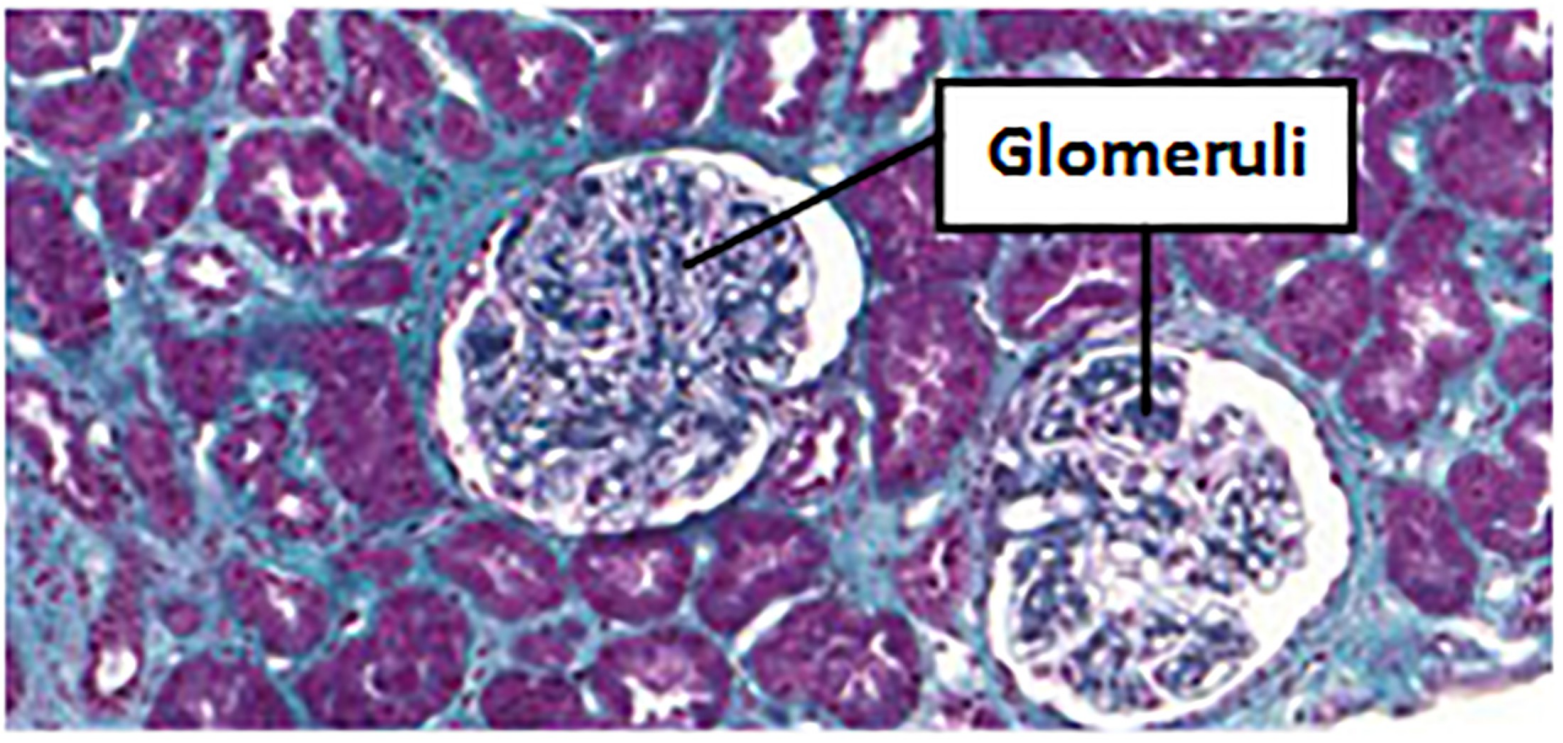
A renal biopsy image with two glomeruli in the GlomDB set [12]. The glomeruli in the image have rich counter-color textures that are composed of stained histological substances.

The GlomDB glomeruli dataset [12] is published for color and texture descriptor evaluation. The image set contains 1976 16-by-16 non-overlapping square patches of textures selected by manual segmentation from 15 Masson’s trichrome stained renal biopsy samples. Among the 1976 sub-images, half textures correspond to glomeruli while the other half are non-glomeruli patches. Fig 4 shows a portion of one renal biopsy image in the GlomDB set, where two glomeruli are observed. Compared to other tissue substances in the renal image that have relatively small color variations, color texture patterns in glomerulus areas are more complicated.

**Fig 5:**
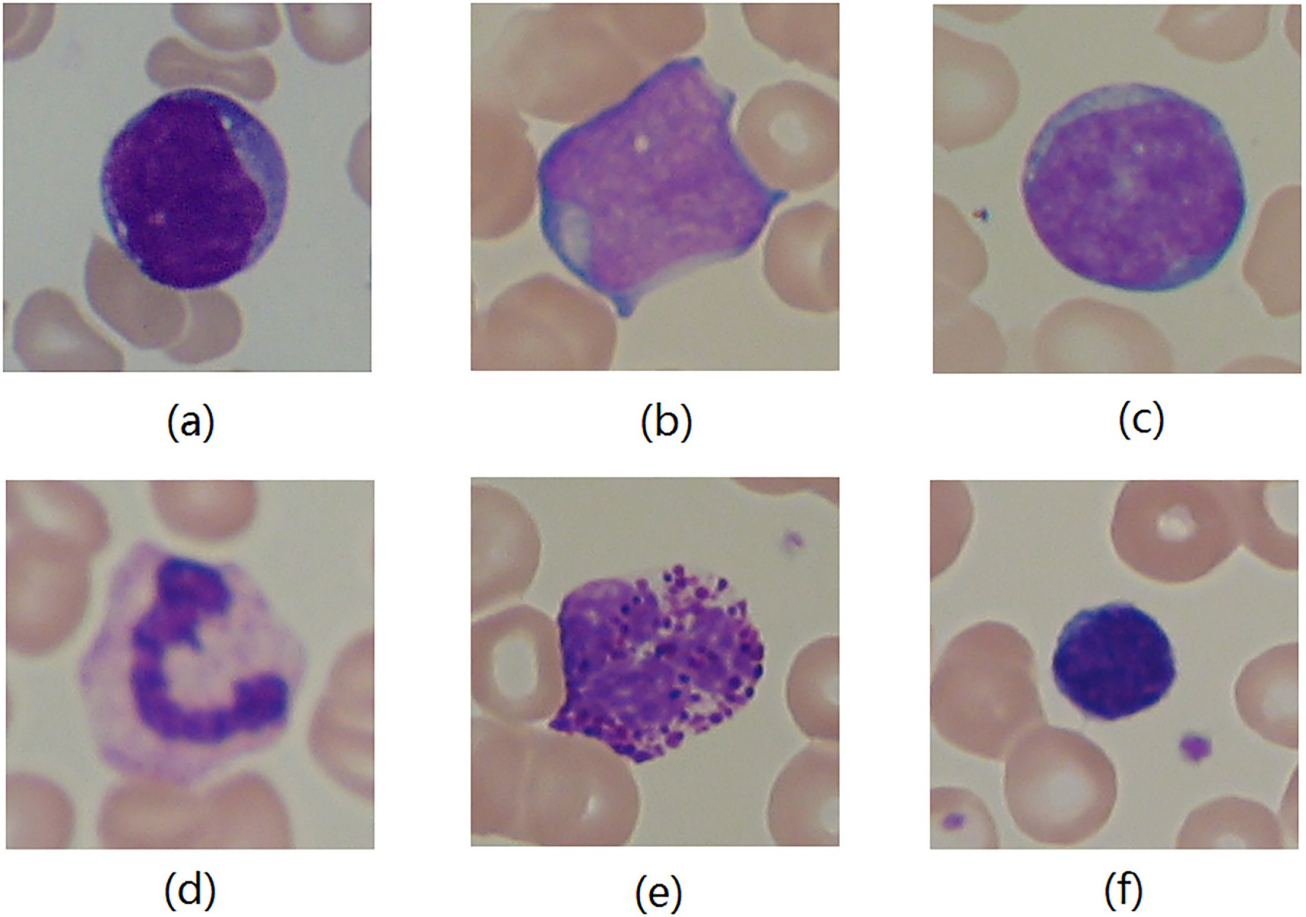
Examples in the ALL-IDB2 image set [42]. (a)-(c) lymphoblasts images and (d)-(f) normal white blood cell images. Compared to the glomeruli image in Fig 4, lymphoblasts images have smoother image color with fewer counter-color textures.

The ALL-IDB2 image set [42] is published for testing the performances of classification systems on blood pathology images. Acute Lymphoblastic Leukemia (ALL) is a serious blood cancer that can be fatal for children. In pathology, identification of blast cells in microscopic images of blood samples is essential in ALL diagnosis. Though morphology characteristics of white blood cells are considered the classical features for distinguishing normal lymphocytes cell and lymphoblasts in ALL images, texture feature LBP is demonstrated having good performance in this classification scenario [9, 10]. Hence, we include this image set in this study to examine our new color texture descriptor. Specifically, the ALL-IDB2 dataset contains 260 cropped area of interest of normal and blast cells from 108 blood sample images generated by Canon PowerShot G5 camera and stored with 24 bit color depth in the RGB format. Among the 260 cell images, 130 images contain lymphoblasts from ALL patients and the rest images have normal white blood cells. Fig 5 shows examples of lymphoblasts and normal lymphocytes cell images in the ALL-IDB2 image set.

### Evaluation protocols

Evaluation of the proposed descriptors is based on pathology images classification in either the GlomDB image set or the ALL-IDB2 dataset. Following the procedure described in previous section, texture features (QDCP, QDCP-LBP, and QDCP/LBP) are extracted and passed to the classifier. To obtain a solid conclusion, fisher’s linear discrinminant (FLD) classifier, the support vector machine (SVM) with a radial basis function (RBF) kernel, and *K*-nearest neighbors (*K*NN) are exploited to evaluate features’ discriminative power. Specifically, we randomly partition the image set into training and testing set following the 10-fold nested cross-validation methodology. In the inner 5-fold validation loop of the training stage, parameters of the proposed descriptor *T*_*O*_ and hyper-parameters of classifiers (*σ* in the RBF kernal and the box constraint for declassification penalty of SVM, and the number of neighbors *k* and the distance metric of *K*NN) are automatically selected to optimize classification accuracy. In the outer testing evaluation loop, the obtained threshold is applied to extract QDCP features which are then passed to the trained classifier.

The agreement between groundtruth and classification results is estimated using two metrics, which are classification accuracy (ACC) and the receiver operating characteristic (ROC) curve analysis. ACC is intuition and easy to compute, representing the probability of a correct classification for a query image. ROC analysis is a more comprehensive measurement than ACC, as it is more statistically consistent in classification evaluation [43]. To quantitatively compare ROC curves of various color texture descriptors, the area under the ROC curve (AUC) is calculated. Both ACC and AUC are in the range of [0, 1], and large values indicate better classification. The 10-fold cross-validation is repeated 10 times, and the statistics of ACC/AUC are summarized based on the 100 performance indexes.

Based on above evaluation procedure, two comparison experiments are performed. In the first experiment, the QDCP descriptor is extracted from the RGB domain, the YCbCr domain, the CIELab domain, and the I1H2H3 domain. The first three color spaces are the most common color spaces used in texture analysis literature, and the I1H2H3 color space is recommended for the Masson’s trichrome stained images in the GLombDB set [12].

In the second experiment, QDCP-based descriptors are compared to prior arts of color texture features. In specific, the grayscale LBP is applied to image luma component only and used as comparison baseline. The non-vector processing methods, ind-LBP and OC-LBP [25], are included in our comparison experiment. Ind-LBP is obtained by concatenating LBP histograms independently extracted from the three channels in the YCbCr color space, and the OC-LBP computes 6 LBP histograms from channel pairs in the YCbCr domain. Finally, 4 state-of-the-art vector-processing based color texture features are examined. Norm-LBP applies the LBP operator to color vector’s magnitude [27]. LCVBP extracts 3 color angular patterns from inter-channel pairs and concatenates them with norm-LBP [14]. LCC-LBP combines the local contrast histogram to LBP descriptor extracted from the image luma component [30]. And LBP-LAP combines 3 angular feature patters computed from inter-channel pairs and 3 LBP histograms from the P1P2P3 space [31]. Note in this work the circular neighborhood Ω_8_ is applied to all descriptors.

## Results and discussion

Quantitative results for each image set are presented and compared to prior works. The sensitivity analysis of QDCP to *T*_*O*_ is examined. All the algorithms and evaluations in this study are implemented in Matlab and accessible to audience.

### Glomerulus texture classification on GlomDB images

Table 2 summarizes glomerulus image classification in the fist comparison experiment that evaluates discriminative power of various color spaces. In this pathology texture analysis image set, QDCP is most descriptive in the YCbCr domain.

**Table 2:** Statistics of glomerulus classification using QDCP extracted from various color spaces (mean±std.).

Color space	FLD		SVM		*K*NN	
	ACC	AUC	ACC	AUC	ACC	AUC
RGB	0.727±0.031	0.794±0.030	0.720±0.084	0.786±0.085	0.720±0.033	0.777±0.037
YCbCr	0.809±0.024	0.892±0.022	0.839±0.023	0.896±0.020	0.843±0.025	0.876±0.043
CIELab	0.706±0.032	0.796±0.032	0.677±0.089	0.755±0.104	0.688±0.035	0.765±0.039
I1H2H3	0.747±0.028	0.828±0.027	0.742±0.086	0.824±0.073	0.738±0.030	0.803±0.021

**Fig 6:**
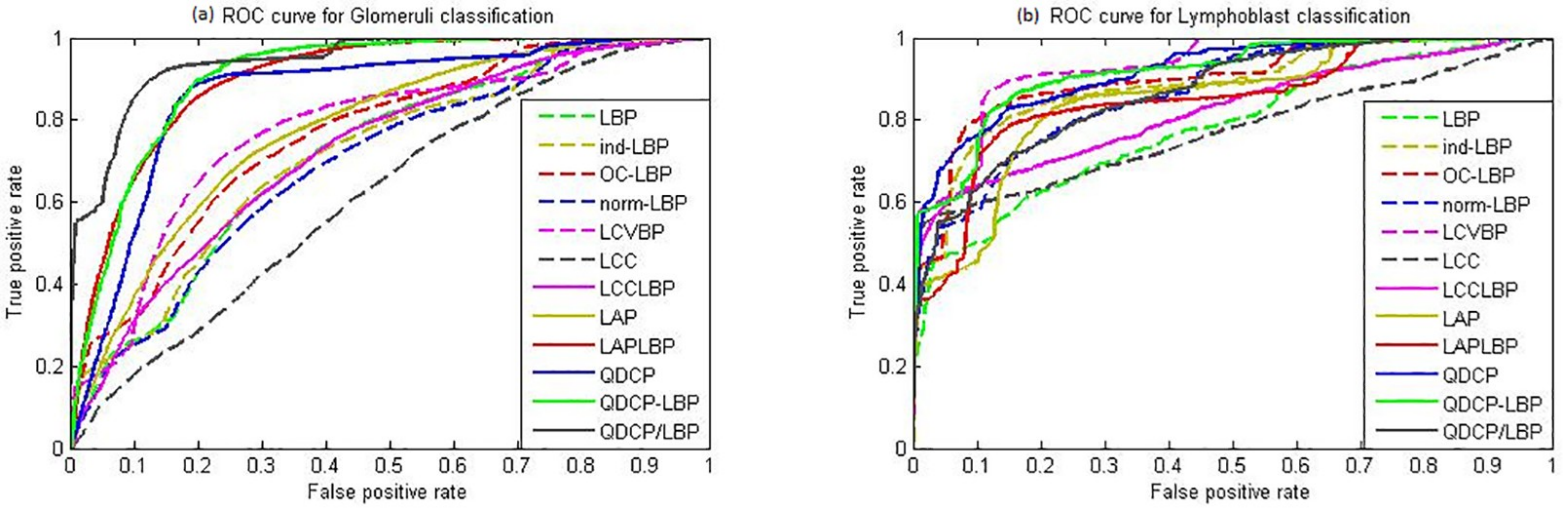
ROC curves for (a)glomerulus classification and (b) lymphoblast classification using various color texture descriptors.

Table 3 summarizes glomerulus image classification using different color texture features over the GlomDB image set, where the top four classification results are highlighted. To visualize the glomerulus classification, ROC curves associated with SVM are depicted in Fig 6(a). Because we observe similar trends in the results associated with FLD and KNN, their ROC curves are omitted here.

**Table 3:** Statistics of glomerulus classification using different color texture descriptors (mean±std.).

Feature set	Dimension	FLD		SVM		*K*NN	
		ACC	AUC	ACC	AUC	ACC	AUC
LBP [8]	36	0.705±0.032	0.775±0.032	0.695±0.040	0.770±0.052	0.691±0.035	0.755±0.043
ind-LBP	3×36	0.716±0.030	0.799±0.031	0.720±0.030	0.792±0.051	0.714±0.034	0.802±0.033
OC-LBP [25]	6×36	0.730±0.028	0.813±0.025	0.730±0.038	0.830±0.052	0.716±0.031	0.803±0.032
norm-LBP [27]	36	0.685±0.031	0.752±0.032	0.689±0.047	0.746±0.051	0.675±0.042	0.733±0.041
LCVBP [14]	4×36	0.758±0.031	0.844±0.026	0.785±0.033	0.853±0.052	**0.792±0.031**	**0.866±0.028**
LCC [30]	256	0.582±0.036	0.619±0.038	0.570±0.041	0.595±0.065	0.583±0.034	0.610±0.045
LCC-LBP [30]	256+36	0.665±0.029	0.726±0.029	0.626±0.040	0.709±0.054	0.641±0.040	0.702±0.048
LAP [31]	3×36	0.715±0.029	0.780±0.030	0.721±0.046	0.795±0.041	0.696±0.017	0.767±0.021
LAP-LBP [31]	6×36	**0.830±0.024**	**0.903±0.018**	**0.813±0.048**	**0.901±0.036**	0.776±0.027	0.861±0.022
QDCP	36	**0.809±0.024**	**0.892±0.022**	**0.839±0.023**	**0.896±0.020**	**0.843±0.025**	**0.876±0.043**
QDCP-LBP	2×36	**0.847±0.023**	**0.919±0.020**	**0.846±0.027**	**0.920±0.024**	**0.838±0.031**	**0.913±0.016**
QDCP/LBP	36^2^	**0.930±0.017**	**0.977±0.010**	**0.897±0.066**	**0.961±0.062**	**0.838±0.032**	**0.920±0.022**

The proposed QDCP-based descriptors (QDCP, QDCP-LBP, and QDCP/LBP) achieve the top performance. This is because QDCP is very descriptive for glomerulus textures that are composed of different stained histological substances as shown in Fig 6(a). In contrast, as color texture descriptors in prior arts are usually based on signal order statistics which are sensitive to small color variation, the resulting features are not reliable in such a scenario. In addition, as expected, QDCP-LBP and QDCP/LBP improve the classification performance compared to QDCP.

Four extra observations are obtained in the GlomDB image set. First, ind-LBP and OC-LBP take color information into account, and thus improve classification performance compared to LBP. Second, norm-LBP and LAP achieves no better result than the comparison baseline, grayscale LBP. For one hand, both norm-LBP and LBP focus on numerical representation of textures associated with color brightness; On the other hand, since LAP is obtained based on ordering of color vectors’ similarity, counter-color texture is prone to being overwhelmed by textures composed by small color variations. Third, LCC has the worst performance, as LCC is an analogy to the local intensity standard deviation in a grayscale image, and less descriptive to color textures. Fourth, both LCVBP and LAP-LBP include LBP and the angular texture patterns extracted from inter-channel pairs, they boost the classification performance compared to LBP.

### Lymphoblast classification in ALL-IDB2 images

Table 4 reports lymphoblast classification in the color space comparison experiment. In the ALL-IDB2 image set, the YCbCr color space and the I1H2H3 color space achieve similar performance. The CIELAB color domain is in the middle, and the RGB color space lags behind.

**Table 4:** Statistics of lymphoblasts classification using QDCP extracted from various color spaces (mean±std.).

Color space	FLD		SVM		*K*NN	
	ACC	AUC	ACC	AUC	ACC	AUC
RGB	0.7658±0.083	0.846±0.082	0.827±0.069	0.888±0.070	0.820±0.071	0.860±0.078
YCbCr	0.869±0.068	0.939±0.045	0.855±0.073	0.927±0.082	0.858±0.080	0.938±0.051
CIELab	0.838±0.065	0.920±0.058	0.811±0.067	0.914±0.082	0.825±0.075	0.916±0.056
I1H2H3	0.860±0.076	0.930±0.051	0.845±0.077	0.911±0.084	0.856±0.079	0.936±0.053

Lymphoblast classification in the ALL-IDB2 image set is reported in Table 5 and Fig 6(b). Again, the top four classification results are highlighted in Table 5. In the ALL-IDB2 dataset, QDCP, QDCP-LBP and LCVBP obtain the top performance for all three classifiers, but the proposed descriptors are more compact than LCVBP, with less than 1/2 size of LCVBP. We also notice that performance of the joint QDCP/LBP is about 8% worse than QDCP-LBP. Theoretically, QDCP/LBP should outperform QDCP-LBP, since QDCP/LBP also maintains the co-occurrence information between QDCP and LBP indexes. However, it should be noted that the information gain of QDCP/LBP is with a penalty of relatively large feature dimension. If the experimental image is not big enough, the resulted QDCP/LBP histogram may be sparse and sensitive to noise and image artifact, which degrades the performance of QDCP/LBP. We believe this is the major reason for the contradictory observation on performance of QDCP/LBP and QDCP-LBP in the glomerulus texture classification and the lymphoblast classification. It should be noted that it is not also the case in practice that the properties of targeting images are known. Hence, we suggest to try both QDCP-LBP and QDCP/LBP for better results.

**Table 5:** Statistics of lymphoblasts classification using different color texture descriptors (mean±std.).

Feature set	Dimension	FLD		SVM		*K*NN	
		ACC	AUC	ACC	AUC	ACC	AUC
LBP [8]	36	0.797±0.071	0.860±0.067	0.783±0.072	0.826±0.064	0.770±0.068	0.816±0.109
ind-LBP	3×36	0.846±0.077	0.910±0.066	0.837±0.078	**0.929±0.054**	0.839±0.079	0.904±0.063
OC-LBP [25]	6×36	0.702±0.084	0.757±0.105	**0.863±0.89**	0.925±0.067	**0.880±0.079**	0.908±0.082
norm-LBP [27]	36	0.797±0.067	0.886±0.058	0.783±0.073	0.887±0.095	0.777±0.074	0.851±0.075
LCVBP [14]	4×36	**0.906±0.054**	**0.959±0.040**	**0.914±0.057**	**0.960±0.034**	**0.894±0.070**	**0.953±0.052**
LCC [30]	256	0.774±0.082	0.768±0.086	0.767±0.069	0.773±0.076	0.774±0.082	0.771±0.075
LCC-LBP [30]	256+36	0.763±0.086	0.829±0.092	0.752±0.067	0.809±0.092	0.753±0.062	0.806±0.091
LAP [31]	3×36	**0.852±0.067**	**0.934±0.047**	0.839±0.081	0.925±0.097	0.858±0.053	0.873±0.065
LAP-LBP [31]	6×36	0.654±0.101	0.689±0.110	0.854±0.096	0.922±0.107	**0.889±0.065**	**0.912±0.065**
QDCP	36	**0.869±0.068**	**0.939±0.045**	**0.855±0.073**	**0.927±0.082**	0.858±0.080	**0.938±0.051**
QDCP-LBP	2×36	**0.895±0.054**	**0.963±0.032**	**0.898±0.074**	**0.951±0.068**	**0.893±0.055**	**0.924±0.060**
QDCP/LBP	36^2^	0.808±0.113	0.897±0.111	0.782±0.103	0.886±0.101	0.765±0.075	0.843±0.072

Different from observations in the GlomDB image set, LBP obtains comparable classification results to other textures. This may be because lymphoblast image color in ALL-IDB2 dataset is much smoother and has less color texture patterns. Another interesting observation is about OC-LBP’s classification results. There is a big performance gap between FLD and the other two classifiers. We believe the reason is that OC-LBP features associated with normal and lymphoblast images are not linearly separable and both SVM and KNN are capable of better handling this nonlinear situation.

### Sensitivity analysis of QDCP against parameter setting

**Fig 7:**
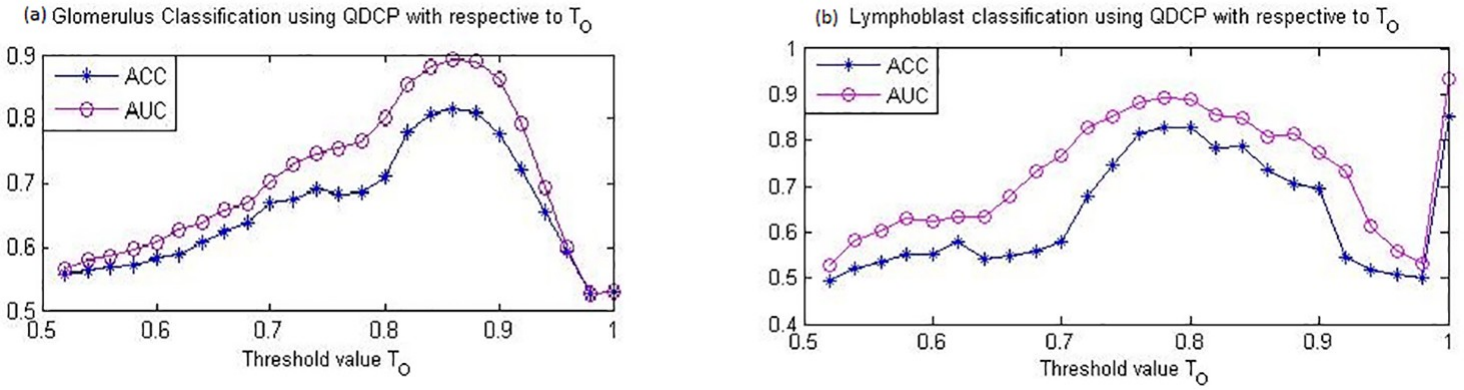
(a) GlomDB image classification and (b) lymphoblast classification based on QDCP with different thresholds.

The effectiveness of the proposed QDCP descriptor depends on the selection of threshold *T*_*O*_ in (5). To examine the sensitivity of QDCP against *T*_*O*_, after representing a query image in the YCbCr domain, we manually vary the value of the parameter when calculating QDCP and feed the obtained QDCP descriptor to a classifier. The average performances in the two testing data sets are report in Fig 7.

Specifically, in Fig 7(a) which corresponds to GlomDB color texture classification, QDCP is most descriptive when threshold *T*_*O*_ is set around 0.85 (which is about 30 degree of color difference). Fig 7(b) depicts the effects of *T*_*O*_ on lymphoblast classification. QDCP obtains strong discriminative power with *T*_*O*_ = 1 and *T*_*O*_ = 0.8, which corresponds to 0 degree and 36 degree of color difference in the orientation domain. An interesting observation is that in lymph cell image classification, *T*_*O*_ = 1 leads to the best performance. This may be because image color in the ALL-IDB2 set is very smooth, and all color change in images are very informative for down-streaming classification.

It is noteworthy that the golmerulus images contains many counter-color content, whereas lymph cell images, in contrast, are relatively dull. On one hand, though the two image sets have distinct image properties, generally, QDCP is descriptive when *T*_*O*_ ∈ [0.8, 0.9]. We believe this observation is applicable to other images. On the other hand, through all experiments in this study, we notice that QDCP-based descriptor, especially QDCP-LBP, have high adaptability to different image sets. We believe that the flexibility of *T*_*O*_ contributes to such adaptability. In other words, different *T*_*O*_ makes the proposed features capable of describing different textures in images. This adaptability is very attractive in image analysis, because usually image properties are not well known beforehand.

## Conclusion

Color texture patterns in pathology images provide insightful information for disease diagnosis. Motivated by the close relation between color diagnosticity and counter-color histological structures in pathology images, this paper introduced a novel compact numerical descriptor, QDCP, for image counter-color texture representation. After projecting image colors to a unit-radius sphere, angular difference between center pixels and their neighbor pixels were summarized in an image-wise histogram. On the basis of QDCP, two new color descriptors, QDCP-LBP and QDCP/LBP, were introduced. Experimentation on publicly available pathology image sets suggested that the proposed descriptors, especially QDCP-LBP, were very descriptive and outperformed state-of-the-art color texture descriptors in terms of discriminative power, computational efficiency and adaptability to different image sets.

Due to the promising performance of the proposed descriptors, our work can be extended in two directions in future. First, observing the high adaptability to different pathology image sets, we want to apply these descriptors to nature texture images, examining their performance in terms of description and adaptability. Second, many algorithms proposed to improve LBP can be adopted by the QDCP-based descriptors. For instance, we may increase the neighborhood dimensionality, introduce multi-thresholding and fuzzy-thresholding descriptors, and examine the uniform patterns on the basis of QDCP.
